# Extensive FUS BBBO to Improve Symptoms of AD

**DOI:** 10.1002/alz70856_103281

**Published:** 2025-12-25

**Authors:** Jin Woo Chang

**Affiliations:** ^1^ Korea University College of Medicine, Seoul, Seoul, Korea, Republic of (South); Korea University College of Medicine, Seoul, Korea, Republic of (South)

## Abstract

**Background:**

Focused ultrasound (FUS)–mediated blood‐brain barrier (BBB) opening is safe and potentially beneficial in patients with Alzheimer's disease (AD) for the removal of amyloid‐beta (Aβ) plaques. However, the optimal BBB opening intervals and number of treatment sessions for clinical improvement remain undefined. Th

**Method:**

In this open‐label prospective study, 6 patients with AD were enrolled from June 2022 to July 2023. FUS mediated BBB opening was performed three times at 2‐month intervals targeting the bilateral frontal lobes. 18F‐florbeta ben positron emission tomography (FBB‐PET) was performed before the first procedure and after the third procedure. Patients were administered neuropsychological and neuropsychiatric evaluations.

**Result:**

All 6 participants completed the study without any acute treatment‐related adverse events. An extensive area of BBB opening (mean 43.1 cm3), more than twice as large as the opening volume (mean 20 cm3) in the authors’ previ ous study, was confirmed by contrast‐enhanced MRI. FBB‐PET scans demonstrated a 14.9‐Centiloid average decrease in Aβ plaques in 4 of the 6 participants (67%), but the Aβ plaques increased in 2 participants after BBB opening, com pared with baseline. No significant changes were observed in the Korean version of the Mini–Mental State Examination in either group. Caregiver‐Administered Neuropsychiatric Inventory scores improved in 5 of 6 participants (83%), indicat ing an improvement in neuropsychiatric symptoms.

**Conclusion:**

This study confirmed the safety and efficacy of more frequent and extensive bilateral frontal BBB opening over multiple sessions in patients with AD. Furthermore, this is the first clinical trial to demonstrate improvement in neuropsychiatric symptoms through BBB opening alone, without concurrent administration of antibody medications.

